# Nucleotide polymorphism affecting *FLC* expression underpins heading date variation in horticultural brassicas

**DOI:** 10.1111/tpj.13221

**Published:** 2016-07-19

**Authors:** Judith A. Irwin, Eleni Soumpourou, Clare Lister, Jan‐Dick Ligthart, Sue Kennedy, Caroline Dean

**Affiliations:** ^1^ John Innes CentreNorwich Research ParkNorwichNR4 7UHUK; ^2^ Bejo Zaden B.V.Trambaan 11749 CZWarmenhuizenthe Netherlands; ^3^ Elsoms Seeds LtdPinchbeck RoadSpaldingPE11 1QGUK

**Keywords:** vernalization, FLC, *Brassica oleracea*, environmental sensitivity, accession numbers KU521322/3

## Abstract

Variation in flowering time and response to overwintering has been exploited to breed brassica vegetables that can be harvested year‐round. Our knowledge of flowering time control now enables the investigation of the molecular basis of this important variation. Here, we show that a major determinant of heading date variation in *Brassica oleracea* is from variation in vernalization response through allelic variation at *FLOWERING LOCUS C*.*C2* (*BoFLC4*). We characterize two alleles of *BoFLC.C2* that are both functional and confer a requirement for vernalization, but they show distinct expression dynamics in response to cold. Complementation experiments in *Arabidopsis thaliana* revealed that the allelic variation results from *cis* polymorphism at *BoFLC.C2*, which quantitatively influences the degree of cold‐induced epigenetic silencing. This results in one allelic variant conferring consistently later heading under both glasshouse and field conditions through reduced environmental sensitivity. Our results suggest that breeding of brassica varieties for commercially valuable variation in heading date has been achieved through the selection of *cis* polymorphism at *FLC*, similar to that underpinning natural variation in *A. thaliana*. This understanding will allow for the selection of alleles with distinct sensitivities to cold and robust heading dates under variable climatic conditions, and will facilitate the breeding of varieties more resistant to climate change.

## Significance Statement

Being able to harvest different broccoli (*Brassica oleracea*) varieties throughout the year is a function of allelic differences at the floral repressor *BoFLC.C2*. Here, we show that epigenetic silencing and reactivation of expression after cold exposure of *BoFLC.C2* are critical factors in this variation. Domestication in horticultural brassicas has therefore exploited similar cis polymorphism at *FLC* to that underpinning natural variation in *Arabidopsis thaliana*.

## Introduction

The transition to reproductive development significantly influences the production of horticultural brassicas: for broccoli and cauliflower the reproductive inflorescence is harvested, whereas for cabbage, kale and Brussels sprouts it is the vegetative tissue that is harvested. Understanding of the molecular control of this developmental transition has come predominantly from studies in *Arabidopsis thaliana*. Mutations in many loci influence flowering time, but the majority of the natural variation in *A. thaliana* flowering time maps to two loci, FRIGIDA (*AtFRI*) and *FLOWERING LOCUS C* (*AtFLC*). Allelic variation at *AtFRI* is the major determinant of vernalization requirement (Johanson *et al*., 2000; Shindo *et al*., 2005), whereas allelic heterogeneity at *AtFLC* explains a large proportion of the variation in vernalization response: namely, how much cold is required to satisfy the vernalization requirement (Shindo *et al*., 2006; Strange *et al*., 2011; Coustham *et al*., 2012; Li *et al*., 2014). Analysis of approximately 1300 *A. thaliana* accessions worldwide identified five major *AtFLC* haplotype groups, defined by non‐coding sequence polymorphism that alters *AtFLC* expression and the degree of epigenetic silencing by cold exposure (Li *et al*., 2014). At least some of these haplotypes appear to confer flowering responses adapted to the local environment (Duncan *et al*., 2015; Li *et al*., 2015a).

The close evolutionary relationship of *A*. *thaliana* and *Brassica oleracea* facilitates the identification of functionally equivalent loci (Parkin *et al*., 2005; Gao *et al*., 2007; Bancroft *et al*., 2011). Analysis of flowering time/heading date in Brassica initially identified quantitative trait loci (QTLs) in *Brassica rapa* and *Brassica napus* in genomic regions that are co‐linear with Arabidopsis chromosome 5, containing *AtFLC*,* AtFY* and *AtCO*, and chromosome 4, containing *AtFRI* (Osborn *et al*., 1997). In *B. oleracea*, the major QTLs for heading date also map to linkage groups C2, C3 and C9, which include this co‐linear region (Rae *et al*., 1999; Axelsson *et al*., 2001; Okazaki *et al*., 2007; Razi *et al*., 2008). A loss‐of‐function mutation in one of these, *BoFLC2*, is associated with earliness in annual cauliflower varieties (Ridge *et al*., 2014), supporting an equivalent role of *FLC* in Brassica and Arabidopsis. Allelic diversity at *AtFRI* has also been associated with heading date variation in *B. oleracea* (Irwin *et al*., 2012; Fadina *et al*., 2013).

To date, there has been little analysis of the variation in vernalization sensitivity between different *B. oleracea* cultivars. Here, through analysis of commercially important variation in broccoli, we show that allelic variation at one *B. oleracea FLC* locus alters vernalization sensitivity. Nucleotide polymorphism between functional *BoFLC.C2* alleles leads to changed vernalization sensitivity under a range of winter conditions. This polymorphism alters the dynamics of *BoFLC.C2* expression, both the cold‐induced *BoFLC* repression and subsequent reactivation on return to warmer conditions. Thus, in the same way as Arabidopsis *FLC cis* polymorphism influences the degree of epigenetic silencing in response to cold. We propose that mining *cis* polymorphism at *BoFLC* will provide a rich source of variation for breeding Brassica crops with an extended season and robust heading dates in response to variable climate.

## Results

### A QTL at *BoFLC.C2* is associated with late flowering under both glasshouse and field conditions

We investigated heading date in four genotypes of purple sprouting broccoli (*B. oleracea* subsp*. italica*; Figure S1a). This variation is used commercially to ensure continuous production of the crop. We chose two genotypes, E5 and E9, for further study. Both have an obligate requirement for cold, but E9 requires longer cold periods than E5 to head. This difference in vernalization response was observed in duplicate field trials at independent UK sites with contrasting winters in Lincolnshire (relatively hard winters) and Cornwall (relatively mild winters; Figure S1b). In these trials the heading date of the E5 parent line significantly differed between years and locations (*P *< 0.001), whereas that of the E9 parent was the same in Lincolnshire in both 2007/8 and 2011/12, and in Cornwall in 2011/12 (*P *= 0.227). Only in Cornwall in 2007/8, with a milder winter in which the mean weekly temperature did not fall below 6°C, was the heading date of the E9 parent significantly later (*P *< 0.001; Figure S1b,c).

**Figure 1:**
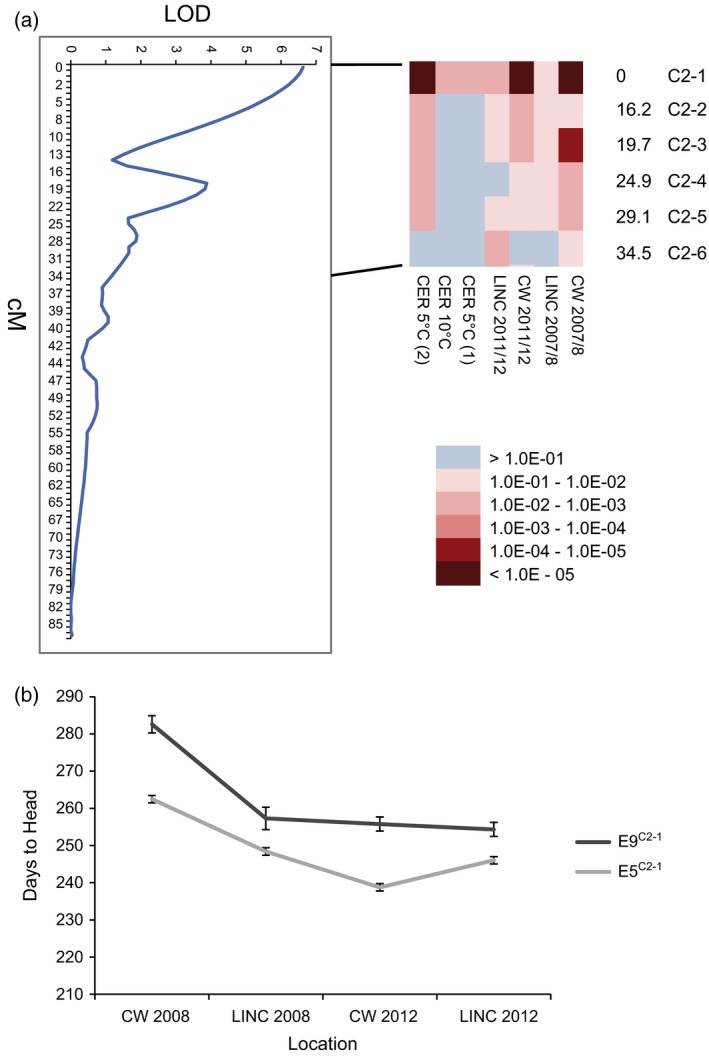
C2 quantitative trait loci (QTLs) analysis for variation in heading date. (a) QTLs mapped to linkage group C2 using single‐marker regression at two independent field sites in Cornwall and Lincolnshire in 2007/8 and 2011/12, and under controlled conditions following a 10‐week vernalization treatment of 5°C or 10°C. Close‐up heat map of climate‐insensitive QTLs mapped to the top of linkage group C2, showing the C2‐1 marker (a marker within the *BoFLC4* gene, AY306124) as that most significantly associated with heading variation (*P *< 1.0E–05) in Cornwall 2007/8 and 2011/12, and under glasshouse conditions following vernalization under controlled conditions at 5°C and 10°C. Key: CW, Cornwall; CER, controlled environment room; LINC, Lincolnshire. Genetic distances are in centimorgans (cM). (b) Heading date variation between DH lines, homozygous for the E5 or E9 alleles at the C2‐1 locus, at the two field sites in 2008 and 2012 showing the consistent difference in heading date between the two parent lines under all conditions. Error bars are standard errors of the mean.

A proprietary Doubled Haploid (DH) mapping population was available from a cross between these genotypes, and we used this to map QTLs controlling vernalization response under both controlled and field conditions. Up to eight QTLs of varying levels of significance were identified, four of which were common across at least four of the seven growth conditions (Figure S1d). One QTL was mapped on all seven occasions. This common QTL, accounting for between 5 and 25% of the heading date variation, mapped to linkage group C2 (Figure 1a). This region of *B. oleracea* chromosome 2 shows conserved synteny with the top of *A. thaliana* chromosome 5, and includes one of the five *B. oleracea* orthologues of *AtFLC*,* BoFLC4* (Lin *et al*., 2005), also known as *BoFLC2* (Ridge *et al*., 2014), and hereafter referred to as *BoFLC.C2*. Individuals homozygous for the E5 and E9 alleles at the marker most significantly associated with heading variation (Figure 1b) show a consistent and robust difference in heading date (on average 14 days) under all conditions tested; with later heading always conferred by the E9 allele (one‐way anova,* P *= 0.0195). Thus, variation at this locus appears to result in differential heading and environmental sensitivity.

### *BoFLC.C2* alleles confer similar phenotypic variation in Arabidopsis

**Figure 2:**
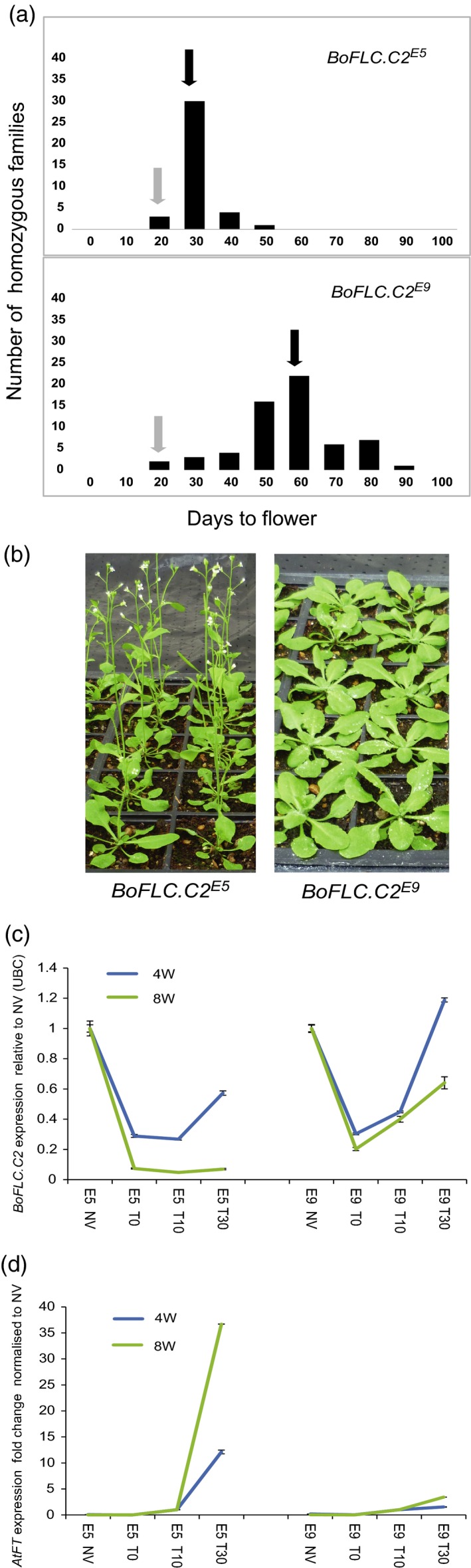
Transgenic Arabidopsis expressing *BoFLC4* ^*E5*^ and *BoFLC4* ^*E9*^. (a) Histogram of the mean flowering time of 48 *BoFLC4* ^*E5*^ and 31 *BoFLC4* ^*E9*^ T_3_ families following 4 weeks of vernalization at 5°C. Black arrows indicate mean flowering time. Grey arrows indicate flowering time of the Col^*FRI*^ ^*flc2*^ control. (b) Differential flowering of *BoFLC4* ^*E5*^ and *BoFLC4* ^*E9*^ homozygous plants. (c) Expression of *BoFLC4* ^*E5*^ and *BoFLC4* ^*E9*^ normalized to expression level before vernalization (NV = 1) at the end of 4 and 8 weeks of cold (T0), and at 10 and 30 days after the return to warm growth conditions (T10 and T30, respectively). Error bars are standard errors of the mean. (d) Expression of *AtFT* normalized to the expression level before vernalization (NV = 1) at the end of 4 and 8 weeks of cold (T_0_), and at 10 and 30 days after the return to warm growth conditions (T10 and T30, respectively). Error bars are standard errors of the mean.

To investigate whether allelic variation at *BoFLC.C2* accounts for this QTL we undertook heterologous complementation experiments. Genomic regions carrying *BoFLC.C2* ^*E5*^ and *BoFLC.C2* ^*E9*^ (6 kb, including the native promoter and terminator) were transformed into the *A. thaliana* genotype Columbia *FRI* *flc2* (Michaels and Amasino, 1999) that carries a loss‐of‐function mutation within *AtFLC*, but has an active *AtFRI*. These experiments aimed to determine whether *BoFLC.C2* could complement the *flc2* mutation, and whether the two alleles would induce different degrees of late flowering. Multiple (48 *BoFLC.C2* ^*E5*^ and 31 *BoFLC.C2* ^*E9*^) independent transgenic plants were generated for each allele and flowering time was analysed in the T_3_ generation. Homozygous *BoFLC.C2* ^*E9*^ transgenic plants flowered consistently later than those carrying *BoFLC.C2* ^*E5*^ (with a mean of 62 ± 1.8 days compared with 34 ± 0.87 days after 4 weeks of vernalization at 5°C; Figure 2a,b). The results of the heterologous complementation therefore support our hypothesis that molecular variation at *BoFLC.C2* contributes to the QTL underlying the variation in vernalization response.

### Two common functional *BoFLC.C2* alleles exist within cultivated *B. oleracea* germplasm

**Figure 3:**
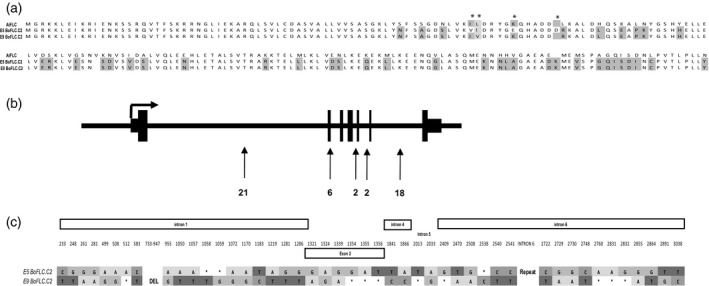
Polymorphism at BoFLC.C2. (a) Amino acid sequence of BoFLC.C2 compared with FLC from Arabidopsis. (b) Ideogram of *FLC* locus showing the location of coding and non‐coding polymorphisms between *BoFLC.C2* ^*E5*^ and *BoFLC.C2* ^*E9*^. All but six (in exon 2) are located in intronic regions, as indicated by black arrows. Numbers indicate the number of polymorphisms in each location. (c) Locations of single‐nucleotide polymorphisms between *BoFLC.C2* ^*E5*^ and *BoFLC.C2* ^*E9*^ relative to the start codon (ATG = +1). Only exons and introns where polymorphisms occur are illustrated. The locations of individual polymorphisms in exon 2 and introns 1, 4, 5 and 6 are indicted by the annotated blocks.

*BoFLC.C2* was previously cloned by Lin *et al*. (2005), and shows similar genomic organization to *AtFLC* with seven exons of similar sizes to those in Arabidopsis and a smaller first intron (approximately 1.1 kb, compared with 3.5 kb in Arabidopsis). We sequenced *BoFLC.C2* from the E5 and E9 genotypes and found that the two alleles encode predicted open reading frames of 198 and 197 residues, respectively (Figure 3a). Three single‐nucleotide polymorphisms in exon 2 confer three non‐synonymous amino acid changes, V66I, I67V (both aliphatic) and E72K. A 3‐bp deletion in E9 results in the loss of an aspartic acid in position 78, compared with the original *BoFLC.C2* identified by Lin *et al*. (2005) and that in E5. An additional 43 polymorphisms are located in introns 1, 4, 5 and 6 (Figure 3b,c), none of which match the polymorphisms reported in the *AtFLC* haplotypes (Li *et al*., 2014).

The high level of non‐coding polymorphism between the *BoFLC.C2* alleles prompted us to sequence a 1300‐bp fragment (covering exon 1 to the end of exon 2) in 20 genotypes from the cultivated *B. oleracea* Diversity Foundation set (described in Irwin *et al*., 2012). These are representative of the six major Brassica crop types (Figure S2a). Nine putatively functional *BoFLC.C2* alleles were identified (Figure S2a). Analysis of polymorphism between the nine allelic classes revealed a network divided into three broad groupings, with *BoFLC.C2* ^*E5*^ and *BoFLC.C2* ^*E9*^ the most frequently occurring alleles within the main two groups, *BoFLC.C2* ^*1‐1*^ and *BoFLC.C2* ^*3‐1*^, respectively (Figure S2b). Once genotypes carrying the previously identified loss‐of‐function single nucleotide polymorphism (SNP) in exon 4 (Okazaki *et al*., 2007; Ridge *et al*., 2014) are removed from the analysis, there is an over‐representation of biennial types carrying the *BoFLC.C2* ^*3‐1*^ allele (Figure S2a).

### *BoFLC.C2* alleles show distinct expression dynamics following cold exposure

**Figure 4:**
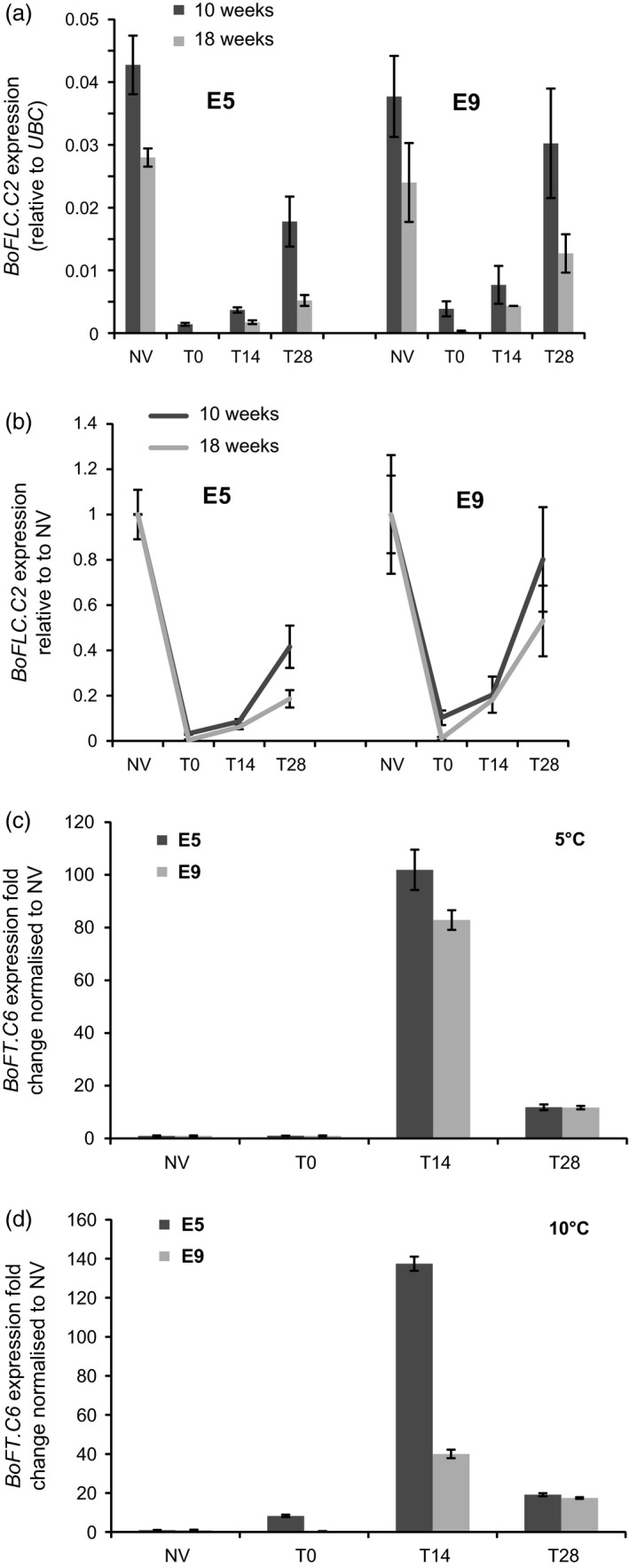
Expression analysis of *BoFLC.C2* and *BoFTC6* in the E5 and E9 genotypes. (a) *BoFLC.C2* expression in the E5 and E9 parental genotypes following 10 weeks (dark grey) and 18 weeks (light grey) of vernalization at 5°C. In all cases *BoFLC.C2* is repressed by cold. On return to warm conditions *BoFLC.C2* appears to reactivate to differing degrees in the two parent lines; however, because of variation between biological replicates these differences were not statistically significant (*P*_v10 weeks_ = 0.238 and *P*_v18 weeks_ = 0.243, respectively). Error bars are standard errors of the mean. (b) *BoFLC.C2* expression expressed relative to the mean before vernalization (NV) starting level for each allele. Relative expression levels are shown for the E5 and E9 parental genotypes following 10 weeks (dark grey) and 18 weeks (light grey) of vernalization at 5°C. Error bars are standard errors. (c) Expression levels of the major *B. oleracea* orthologue of the floral promoter *FLOWERING LOCUS T* (*FT*) on chromosome C6 after vernalization at 5°C. The graph shows *BoFT.C6* fold change relative to the starting level NV following 10 weeks of cold. *BoFT.C6* is induced to a higher level at 14 days after the return to warm conditions in E5 (dark grey), compared with E9 (light grey, *P *< 0.001). Error bars are standard errors. (d) Expression levels of the major *Brassica oleracea* orthologue of the floral promoter *FLOWERING LOCUS T* (*FT*) on chromosome C6 after vernalization at 10°C. The graph shows *BoFT.C6* fold change relative to the starting level NV following 10 weeks of cold. *BoFT.C6* is induced to a higher level at 14 days after the return to warm conditions in E5 (dark grey), compared with E9 (light grey, *P *< 0.001). Error bars are standard errors.

To determine how polymorphism at *FLC* leads to the phenotypic differences observed, we analysed *BoFLC.C2* ^*E5*^ and *BoFLC.C2* ^*E9*^ expression in the *B. oleracea* E5 and E9 genotypes by quantitative real‐time RT‐PCR. Both *BoFLC.C2* alleles are repressed by 10 weeks of vernalization at either 5°C or 10°C (Figures 4a,b and S3a). On return to warm conditions, however, the alleles reactivate to differing degrees.

To investigate these differential expression dynamics further, we analysed *A. thaliana* transgenic lines homozygous for the *BoFLC.C2* ^*E5*^ and *BoFLC.C2* ^*E9*^ alleles. Three *BoFLC.C2* ^*E5*^ and *BoFLC.C2* ^*E9*^ T_3_ homozygous transgenic lines were selected with flowering times that matched the mean of all the transgenic lines (Figure 2a). These *BoFLC.C2* ^*E5*^ and *BoFLC.C2* ^*E9*^ lines showed differences in flowering time after 4, 6 and 8 weeks at 5°C, consistent with the *BoFLC.C2* ^*E9*^ allele being less sensitive to cold exposure (Figure S4a, b). Following 4 weeks at 5°C the expression of both the *BoFLC.C2* ^*E5*^ and *BoFLC.C2* ^*E9*^ alleles reactivated after plants were returned to warm conditions; however, following 8 weeks at 5°C, expression of the *BoFLC.C2* ^*E5*^ allele was almost completely repressed when plants were returned to the warm, whereas expression of the *BoFLC.C2* ^*E9*^ allele reactivated (Figures 2c and S5). These data support the hypothesis that *cis* polymorphism at *BoFLC.C2* accounts for the differential silencing dynamics between the two alleles.

Given the differential responses of the *BoFLC.C2* alleles in the Arabidopsis transgenic lines, we analysed *BoFLC.C2* expression in the E5 and E9 Brassica parents following an 18‐week vernalization treatment at 5°C. This confirmed that even after this extended cold exposure the two alleles show different silencing dynamics: *BoFLC.C2* ^*E5*^ reactivated to approximately 18% of the expression level found in non‐vernalized plants, whereas *BoFLC.C2* ^*E9*^ reactivated to approximately 52% of that observed in non‐vernalized plants (Figure 4a).

### Activation of *BoFT* expression is attenuated in E9

We assayed the expression of the two *B. oleracea FLOWERING LOCUS T* loci (*BoFT.C2* and *BoFT.C6*) in the E5 and E9 genotypes (Figures 4c,d and S3b). It has previously been reported that only one of these, *BoFT.C6*, is expressed in *B. oleracea* and *B. napus* (Wang *et al*., 2012), and that loss‐of‐function mutations in this gene result in late flowering in *B. napus* (Guo *et al*., 2014). We found increased expression of both copies of *BoFT* 14 days after plants were returned to warm conditions, however, this was transient, decreasing again by 28 days. Significantly, this increase in expression of *BoFT* was not as high in E9 as in E5 after vernalization at 10°C (Figures 4d and S3b). Similarly, *BoFLC.C2* ^*E9*^ induced *AtFT* more slowly than *BoFLC.C2* ^*E5*^ in the *A. thaliana* transgenic lines (Figure 2d). These data are consistent with the reduced silencing of *BoFLC.C2* ^*E9*^ influencing the level of *FT* induction, which in turn influences heading date.

## Discussion

A requirement for vernalization defines whether a plant overwinters before flowering – a winter annual, biennial or perennial – or adopts a summer annual or rapid‐cycling reproductive strategy, which in some environments enables multiple generations a year. As such, vernalization requirement and the ability to fine‐tune the cold‐temperature sensitivity of vernalization provides key variation that plant breeders have used and will need in the future to develop varieties in an era of climate change. Here, we provide evidence for how *cis* polymorphisms between functional alleles at one of the *B. oleracea FLC* loci (*BoFLC.C2*) regulate the cold‐sensitivity requirement of vernalization. Our analysis identified *BoFLC.C2* as a candidate for a major QTL for heading date variation after different exposures to cold. Heterologous complementation analyses in *A. thaliana* then showed that the differential expression dynamics of the two *BoFLC.C2* alleles post‐vernalization were recapitulated in the transgenic lines. Thus, *cis* variation between two functional *BoFLC.C2* alleles confers quantitative variation in heading date, via an influence on the epigenetic silencing of *FLC* expression. Therefore, the two *BoFLC.C2* alleles show differential environmental sensitivity, an important factor in commercial broccoli production (Figures S1 and S4).

Which of the *cis* polymorphisms is responsible for *BoFLC.C2* expression variation is yet to be determined. The fact that the exon‐2 polymorphisms do not appear to segregate with crop type (Figure S2a) points to non‐coding intronic polymorphism at *BoFLC.C2* underpinning quantitative variation in cold‐induced *BoFLC* silencing and reactivation. There are therefore parallels between this molecular variation and the natural variation in vernalization response in *A. thaliana* (Coustham *et al*., 2012). The conservation of *COOLAIR* (Swiezewski *et al*., 2009), the *AtFLC* antisense RNA in *Arabis alpina* (Castaings *et al*., 2014) and in *B. rapa* (Li *et al*., 2015b), also points to strong commonality in the regulation of *FLC* across the Brassicaceae. Non‐coding sequence polymorphism has recently been shown to define a small number of major *AtFLC* haplotype groups varying in *AtFLC* expression and degree of epigenetic silencing (Li *et al*., 2014). These haplotypes can be grouped as RV (rapid vernalization) and SV (slow vernalization) types. Accessions within the SV groups require longer periods of cold before *AtFLC* is epigenetically silenced, and in at least two cases (SV2 and SV4) appear to flower at a similar time following the same number of weeks of cold at temperatures ranging between 2°C and 12°C (Duncan *et al*., 2015). This similarity in heading date across a range of different growth conditions suggests parallels with the observed decreased environmental sensitivity of the *BoFLC.C2* ^*E9*^ allele.

Repression of the different functional *BoFLC.C2* alleles activates the expression of *B. oleracea FLOWERING LOCUS T*, as previously reported for annual cauliflower under field conditions (Ridge *et al*., 2014). *BoFT.C2* and *BoFT.C6* were both activated once FLC expression was reduced, but to very different levels (Figures 4c,d and S3b), and only transiently. This transient activation was not observed in the Arabidopsis transgenic lines (Figures 2d and S6). The reason for this difference is unknown. In Arabidopsis, *AtFLC* is thought to act as an inhibitor of *AtSOC1* and *AtFT* by binding to *cis*‐regulatory elements in intron 1 of *AtFT* and the *AtSOC1* promoter (Helliwell *et al*., 2006; Searle *et al*., 2006). It may be that *FLC* in the monocarpic *A. thaliana* becomes functionally irrelevant once this repression has been released and *AtFT* is activated. The expression dynamics of *BoFT.C2* and *BoFT.C6* resemble those of *AhFT* in *Arabidopsis halleri*,* a* perennial relative of *A. thaliana*, where *AhFT* expression increases after prolonged cold, but is then repressed in subsequent warm temperatures concomitant with the reactivation of *AhFLC* (Aikawa *et al*., 2010). A similar pattern of expression is seen in the orthologue of *AtFT* in perennial poplar (*PdFT2*; Hsu *et al*., 2011) that is associated with reproductive onset and bud break; *PdFT2* transcription was promoted by warm temperatures and long‐day conditions, and was repressed by cold temperatures and short days. The parallels in the quantitative, inverse correlation of *FLC* and *FT* expression between *B. oleracea* and *A. halleri* reinforce the previously reported view that *B. oleracea* crops are derived from a wild ancestor with a short‐lived perennial life cycle (Raybould *et al*., 1999). They also support the conclusion that quantitative differences in *BoFLC* silencing are the cause of the heading date variation between the E5 and E9 *BoFLC.C2* alleles.

This analysis provides another important example of *cis*‐regulatory variation driving a major adaptive trait under both natural selection and domestication. Evidence has previously been reported in *Zea mays* (maize) of domestication resulting in a variation bottleneck caused by the removal of *cis*‐acting polymorphisms, as a consequence of manmade selection (Hufford *et al*., 2012). In the case of *BoFLC* this *cis* variation opens up the possibility of exploiting allelic diversity for predictive breeding for vernalization response and cold sensitivity, aiming for the consistent, continuous scheduling of brassica vegetables. *BoFLC.C2* ^*E9*^ is an example of a strong *BoFLC* allele that is less sensitive than others to cold temperatures. With increased variability in winter temperatures, mining germplasm for allelic variation at this and other Brassica *FLC* loci should help to breed less environmentally sensitive varieties and reduce crop losses.

## Experimental Procedures

### Plant material and growth conditions

*Arabidopsis thaliana* seeds were grown as described previously (Irwin *et al*., 2012). Flowering time was recorded as the number of days to flowering, determined when the inflorescence stem was 3‐cm tall.

*Brassica oleracea* plants were germinated in long‐day glasshouse conditions (16‐hour photoperiod) at 18°C (lit by 400‐W HQI metal halide lamps where supplementary lighting was required). Seedlings were pricked out after 11 days and grown for 70 days in experiment 1 and 90 days in experiment 2, so the plants were no longer in the juvenile phase and were responsive to vernalization treatment. Plants were then vernalized for 10 or 18 weeks under a short‐day photoperiod at 5°C or 10°C in a controlled environment room. Plants were transferred to warm conditions in April 2010 and 2011, potted on into 2‐L pots and grown in an unheated glasshouse with a minimum 14‐hour natural photoperiod. Plants were scored for buds visible at the apex, apical head at approximately 12 mm (the size of a 10‐pence coin in the UK) and date of opening of first flower.

### Field experiments

A total of 120 and 82 Doubled Haploid (DH) lines from the proprietary mapping population were transplanted into the field at independent sites in Cornwall (50°12′45.97″N, 5°17′41.19″W) and Lincolnshire (52°47′13.52″N, 0°09′5.08″E) in three randomized, replicated blocks in July 2007 and 2011, respectively. The plots were netted to prevent damage from rabbits and pigeons. Plants were scored for buds visible at the apex, apical head at approximately 12 mm (the size of a 10‐pence coin in the UK) and date of opening of first flower.

### QTL analysis

Single‐marker regression analysis in which each marker was individually regressed against the phenotypic data was conducted in genstat (Payne *et al*. 2009) using mean values for heading date. Potential QTL locations were estimated as genomic regions where loci were statistically significant at *P *< 0.05, with the marker with the highest *F* probability in the analysis defined as the QTL peak and therefore closest to any putative candidate genes.

### Functional analysis of BoFLC.C2 alleles

A 6‐kb fragment containing *BoFLC.C2* was isolated from genomic DNA of lines E5 and E9 by PCR with primers FLC4‐*Xho*I‐F and FLC4‐*Xho*I‐R, using *PfuUltra* II Fusion HS DNA Polymerase (Agilent Technologies, http://www.agilent.com) following the manufacturer's conditions, and was then sequenced (primers are given in Table S1). The constructs were ligated into binary vector pSLJ1711 (a gift from Prof. Jonathan Jones, Jones *et al*., 1992). The constructs were transferred into *Agrobacterium* by triparental mating and transformed into *A. thaliana* accession Columbia *FRI flc2* by a floral‐dipping method (modified from Bechtold *et al*., 1993). T_1_ transformants were isolated by selection for Kanamycin resistance. T_2_ and T_3_ seeds were collected, homozygous lines were identified and flowering time was determined by ‘days to flower’, excluding the period of vernalization treatment.

### Sequencing *BoFLC.C2* in BolDFS

The *B. oleracea* Diversity Foundation set (BolDFS) is a core collection of lines that represent the genetic variation across the morphologically diverse crops of this species (http://www.brassica.info/resource/plants/diversity_sets.php). DNA was isolated as described in Irwin *et al*. (2012). A 1300‐bp fragment of *BoFLC.C2* was then amplified from DNA of 20 genotypes of the BolDFS by PCR with primers FLC4_F13 and FLC4_R13, using AMPLITAQ GOLD TAQ DNA Polymerase (Life Technologies Ltd, now ThermoFisher Scientific, http://www.thermofisher.com). Sequences were aligned using AlignX in Vector NTI (Invitrogen) and analysed in splitstree4 (Huson and Bryant, 2006) using the median‐joining method (Bandelt *et al*., 1999).

### Expression analysis

Total RNA from three biological replicates was extracted from leaf material sampled at the same time of day as described previously (Etheridge *et al*., 1999; Box *et al*., 2011). RNA was DNase‐treated to remove contaminating DNA using an Ambion Turbo DNase kit according to the manufacturer's instructions. cDNA was synthesized using the Invitrogen Superscript^™^ III First Stand Synthesis System (18080‐051; Invitrogen, now ThermoFisher Scientific) and analysed by qPCR on a LightCycler 480 II instrument (Roche, http://www.roche.com), using LightCycler 480 SYBR Green I Master mix (Roche Diagnostics, http://www.roche-diagnostics.com). *BoFLC.C2* mRNA accumulation was assayed with primers BoFLC.C2‐F and BoFLC.C2‐R. Expression was normalized using an orthologue of the Arabidopsis gene *UBC* (*At5g25760*, GENBANK EU593895), with primers UBC‐F and UBC‐R. *BoFT.C2* and *BoFT.C6* were similarly assayed using the following primer combinations: BoFT.C2‐F1 and BoFT.C2‐R1; BoFT.C6‐F2 and BoFT‐C6‐R2.

Expression analysis in Arabidopsis transgenics was conducted on pooled samples of homozygous T_3_ seedlings from two T_3_ lines derived from three independent T_2_ lines selected from the median of the flowering time distribution of 48 E5 and 31 E9 T_2_ families, respectively. RNA was extracted for each pool, DNase‐treated, and cDNA synthesis conducted and analysed by qPCR, as described above. *BoFLC.C2* expression was assayed using the BoFLC‐F and BoFLC.C2‐R primers. Expression was normalized using the Arabidopsis gene *UBC* (*At5g25760*) with primers UBC21_UPL_F and UBC21_UPL_R, and PP2A (*At1g13320*), PP2A_F2 and PP2A_R2. Expression of endogenous Arabidopsis *FT* was assayed with primers FT_UPL#138_F and FT_UPL#138_R. Primers are listed in Table S1.

## Supporting information


**Figure S1.** Heading date variation in purple sprouting broccoli.

**Figure S2.** Allelic variation at BoFLC.C2.

**Figure S3.** Expression analysis in E5 and E9 parent lines.

**Figure S4.** Flowering time of Arabidopsis *BoFLC.C2* transgenics.

**Figure S5.** Expression of *BoFLC.C2* alleles in transgenic Arabidopsis.

**Figure S6.** Expression of *AtFT.C2* in transgenic Arabidopsis.

**Table S1.** Primers used in this study.
