# Supplementary figures and images for: Nucleotide polymorphism affecting FLC expression underpins heading date variation in horticultural brassicas
Source: Plant J. 2016 Jul 19;87(6):597–605. doi: 10.1111/tpj.13221 (PMC5053238; doi:10.1111/tpj.13221)

Supplementary figure 1.

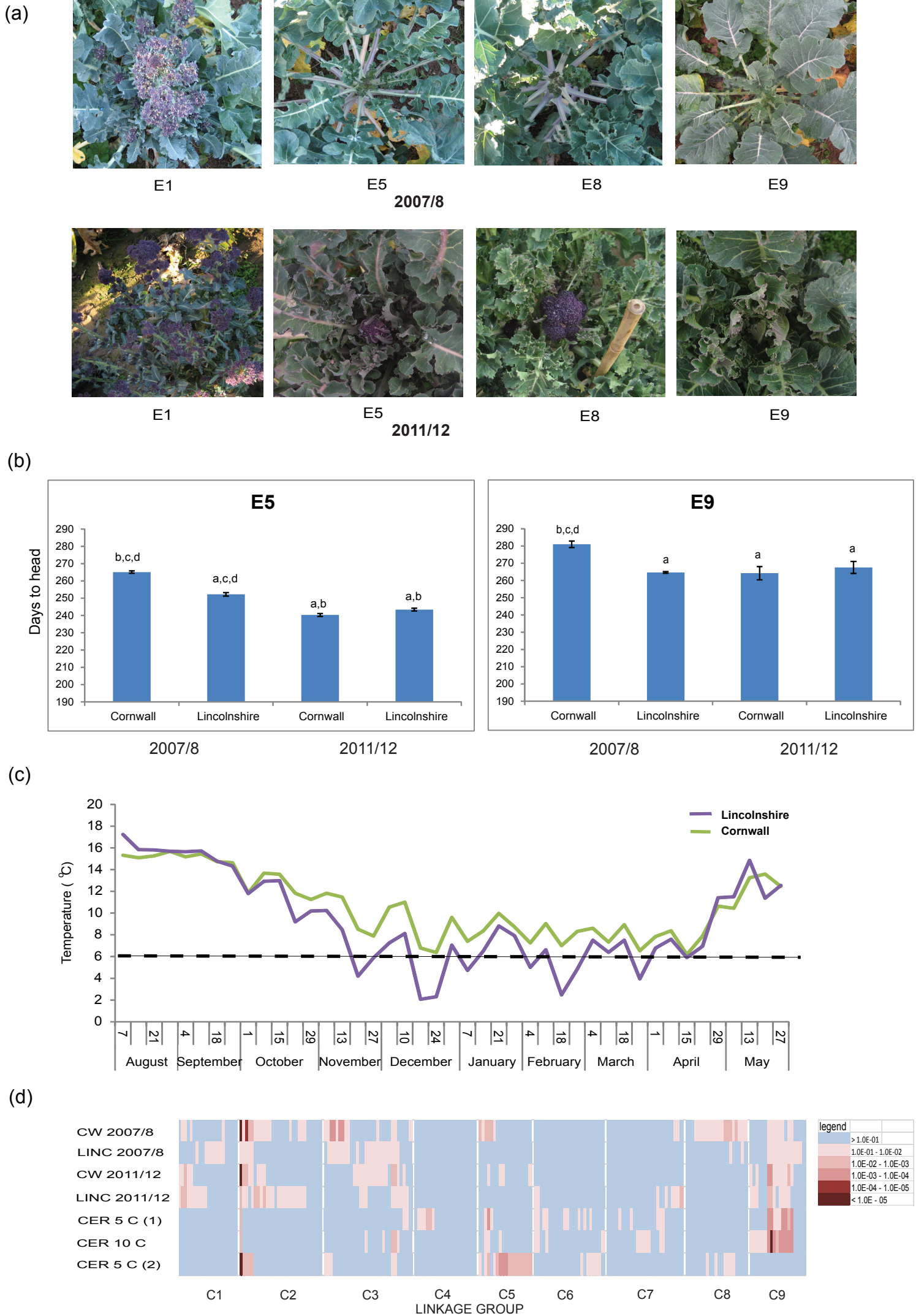

Supplement: Supplementary file 1 — Figure S1. Heading date variation in purple sprouting broccoli. [file TPJ-87-597-s001.pdf]

Supplementary Figure 3

(a)

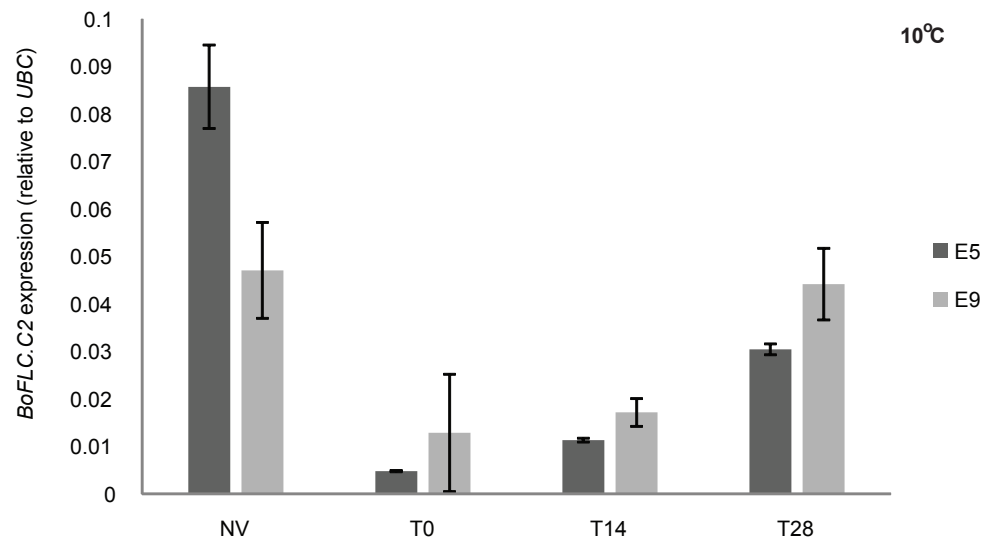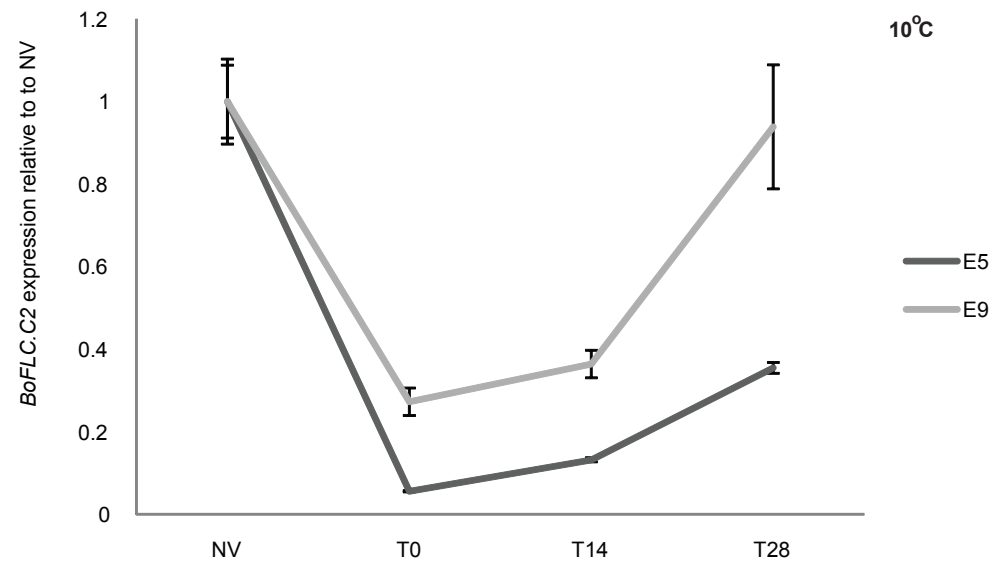

(b)

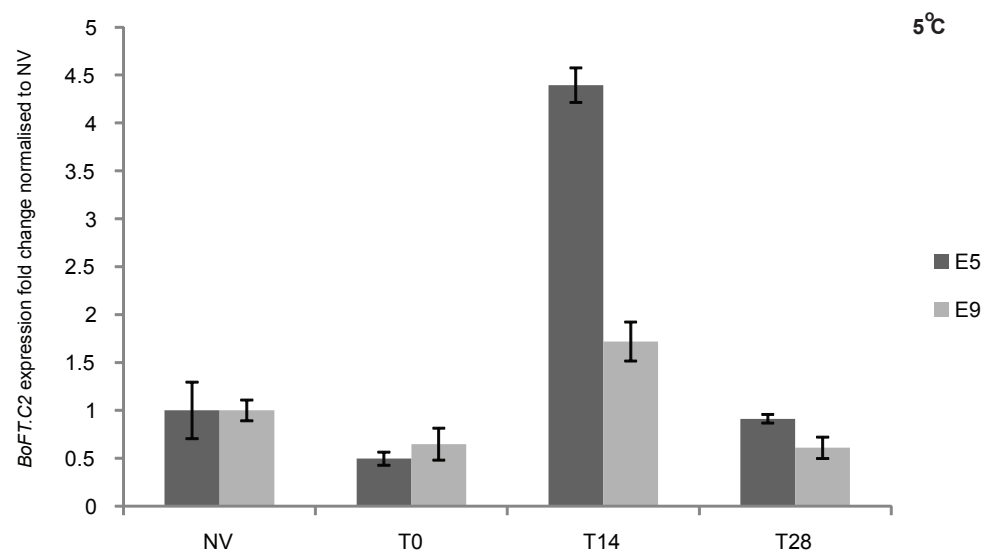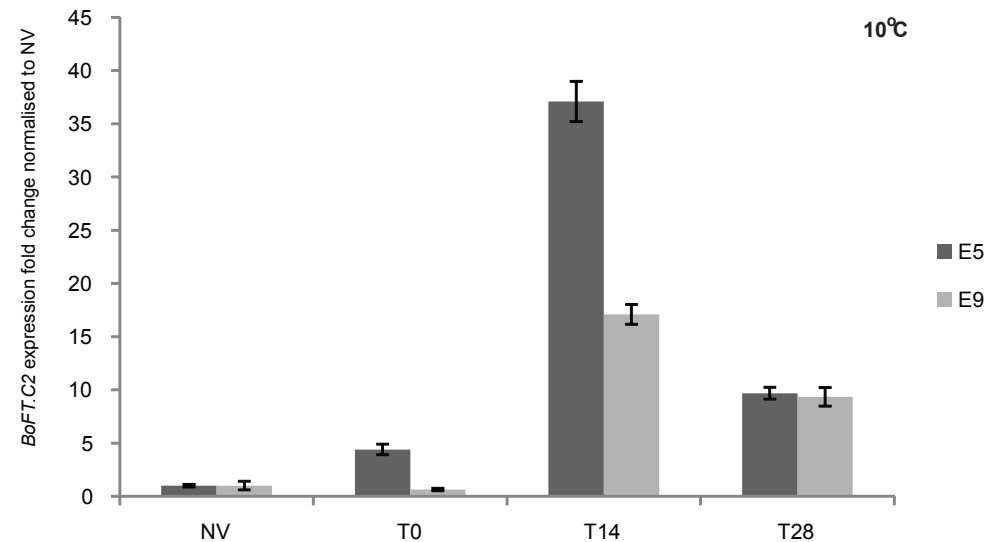

Supplement: Supplementary file 3 — Figure S3. Expression analysis in E5 and E9 parent lines. [file TPJ-87-597-s003.pdf]

Supplementary figure 4.

(a)

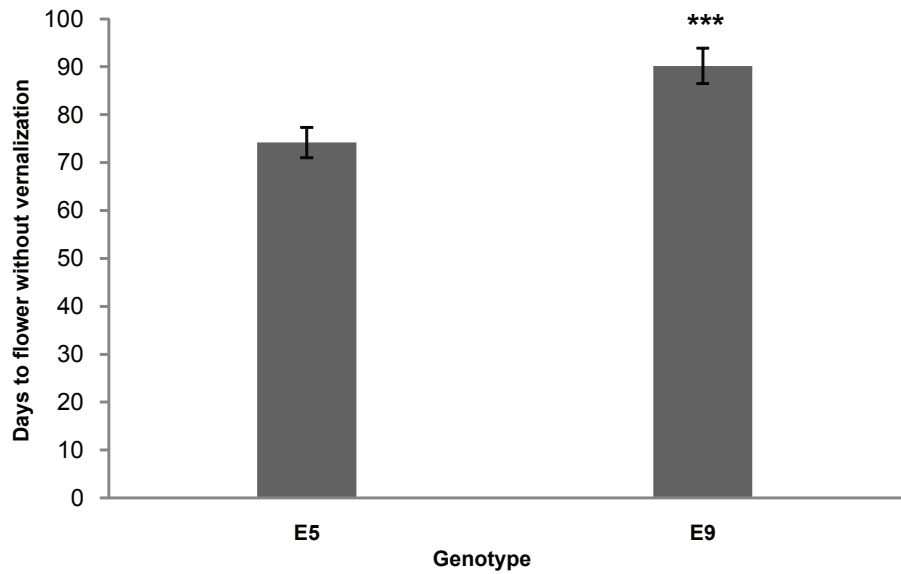

(b)

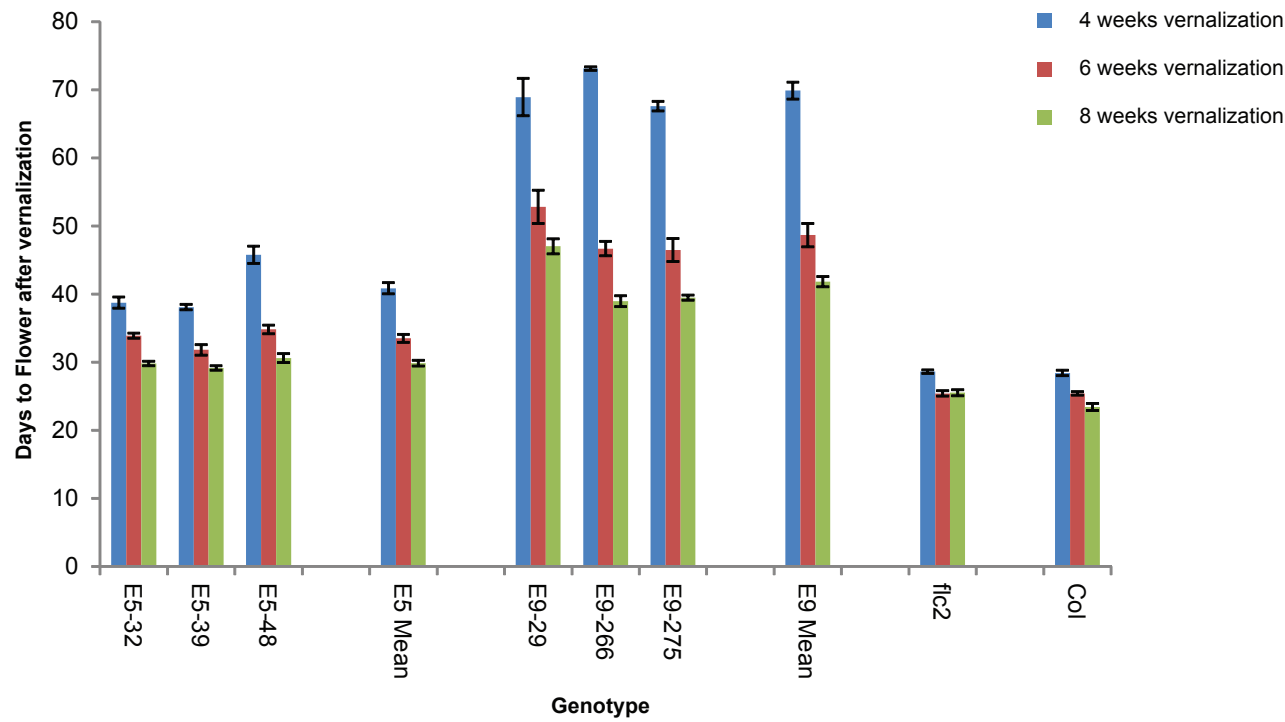

Supplement: Supplementary file 4 — Figure S4. Flowering time of Arabidopsis BoFLC.C2 transgenics. [file TPJ-87-597-s004.pdf]

Supplementary Figure 5

4 weeks vernalization

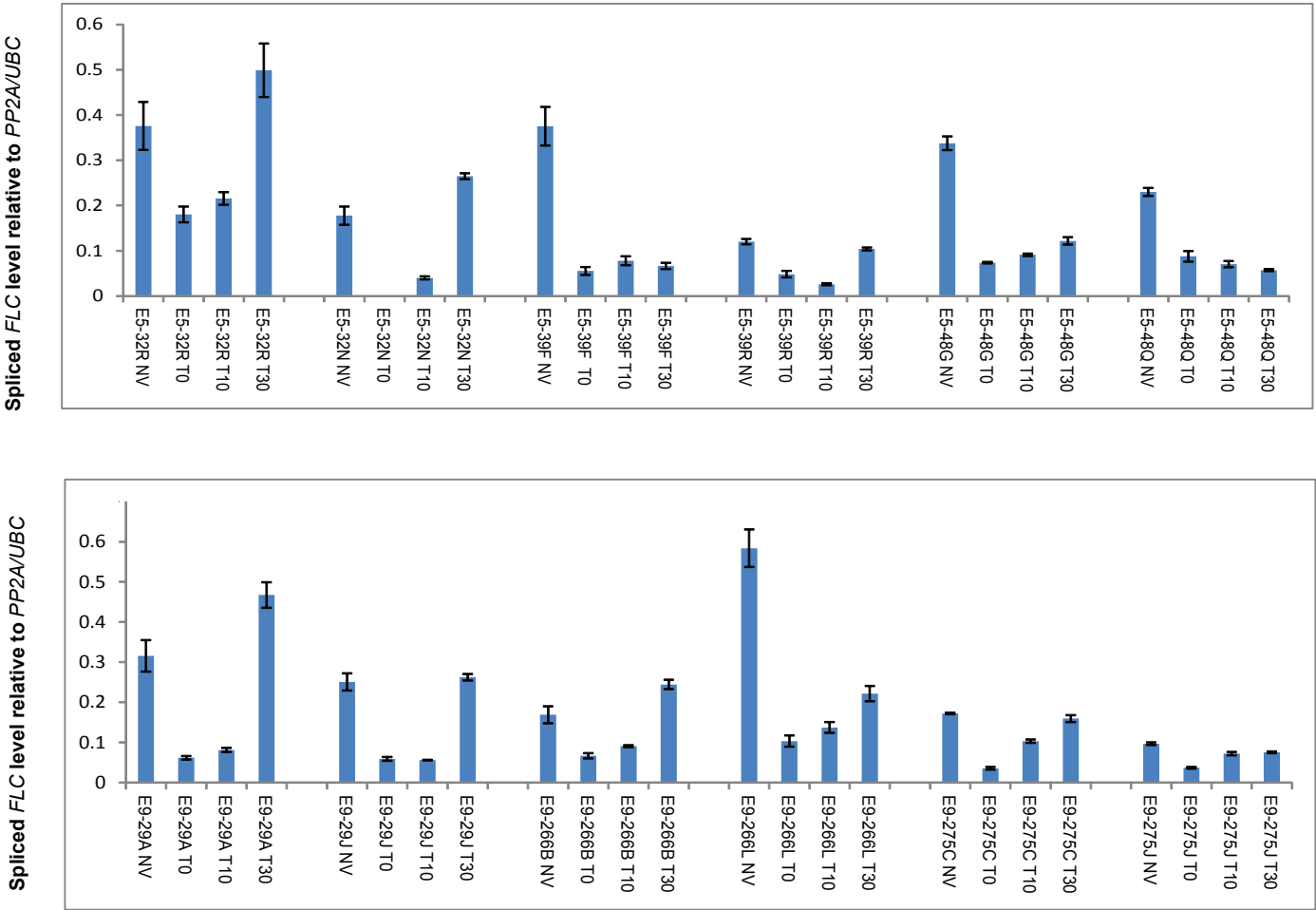

8 weeks vernalization

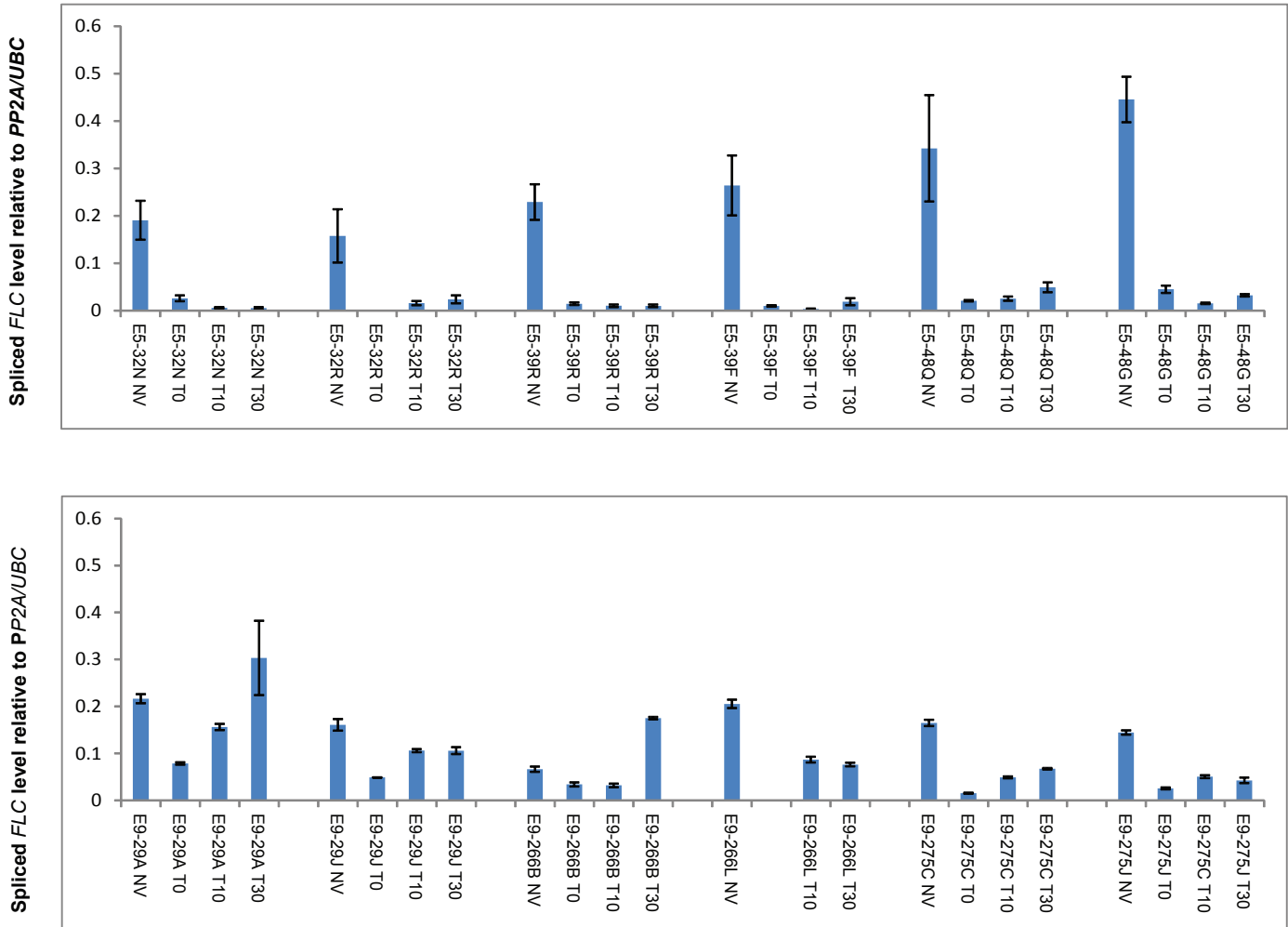

Supplement: Supplementary file 5 — Figure S5. Expression of BoFLC.C2 alleles in transgenic Arabidopsis. [file TPJ-87-597-s005.pdf]

Supplementary figure 6.

4 weeks vernalization

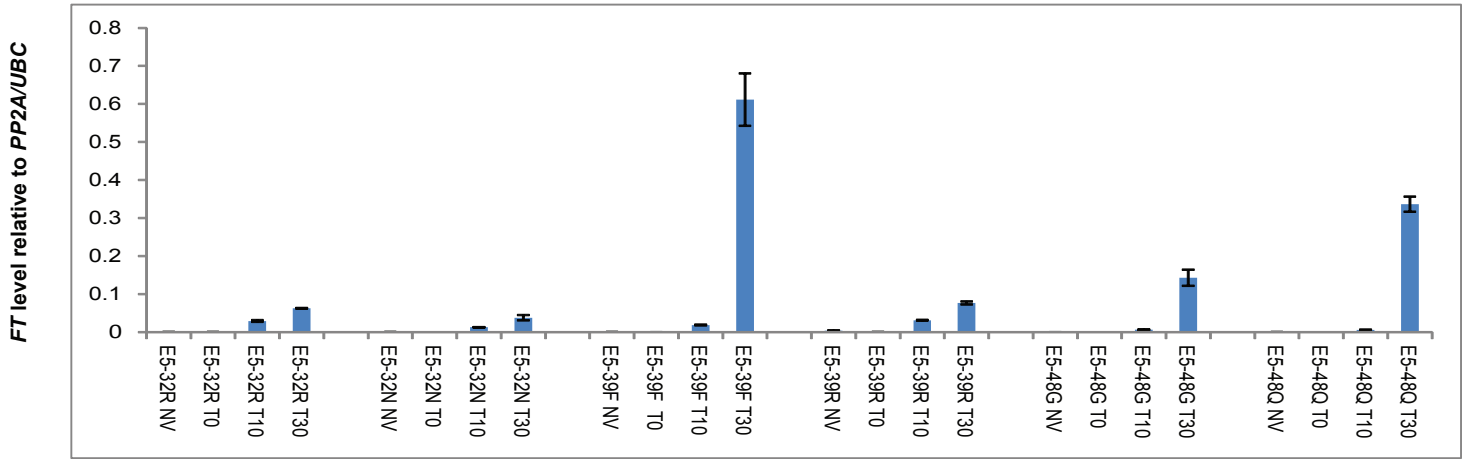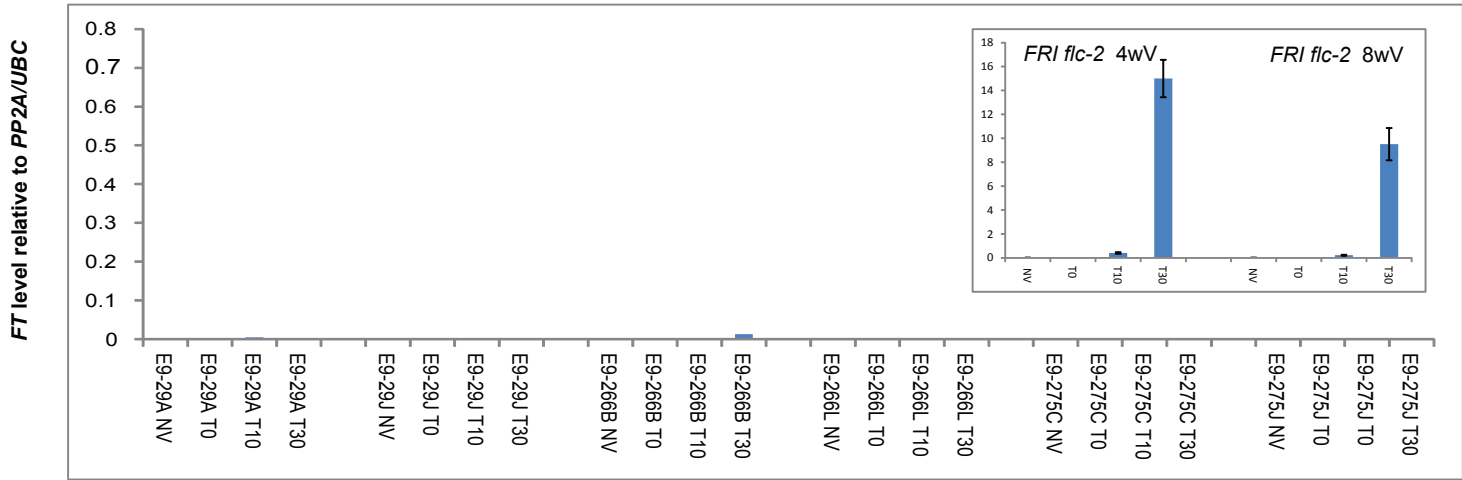

8 weeks vernalization

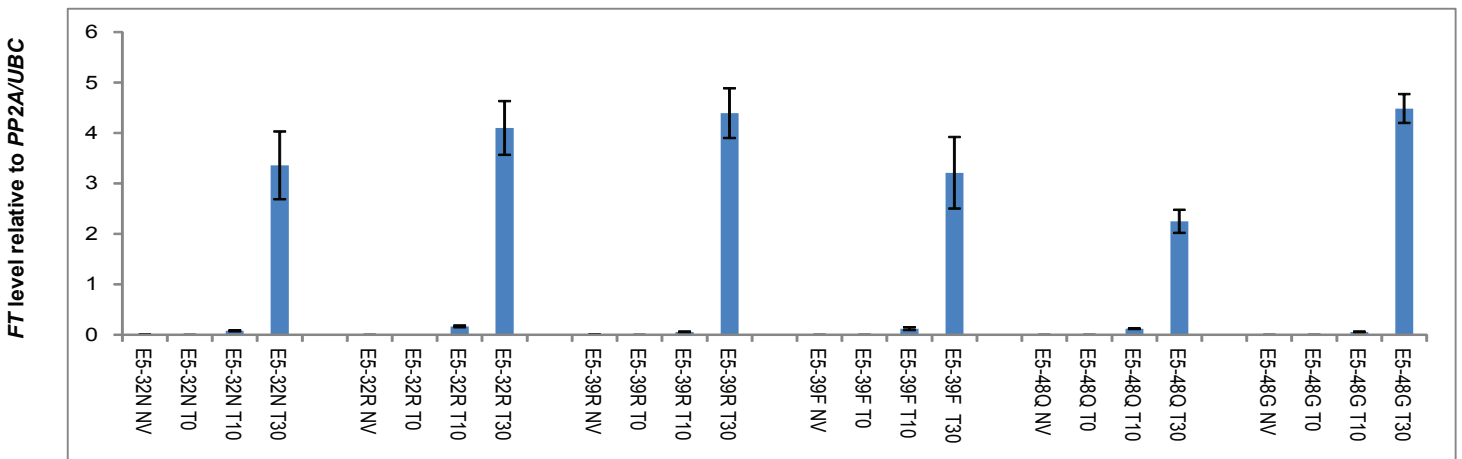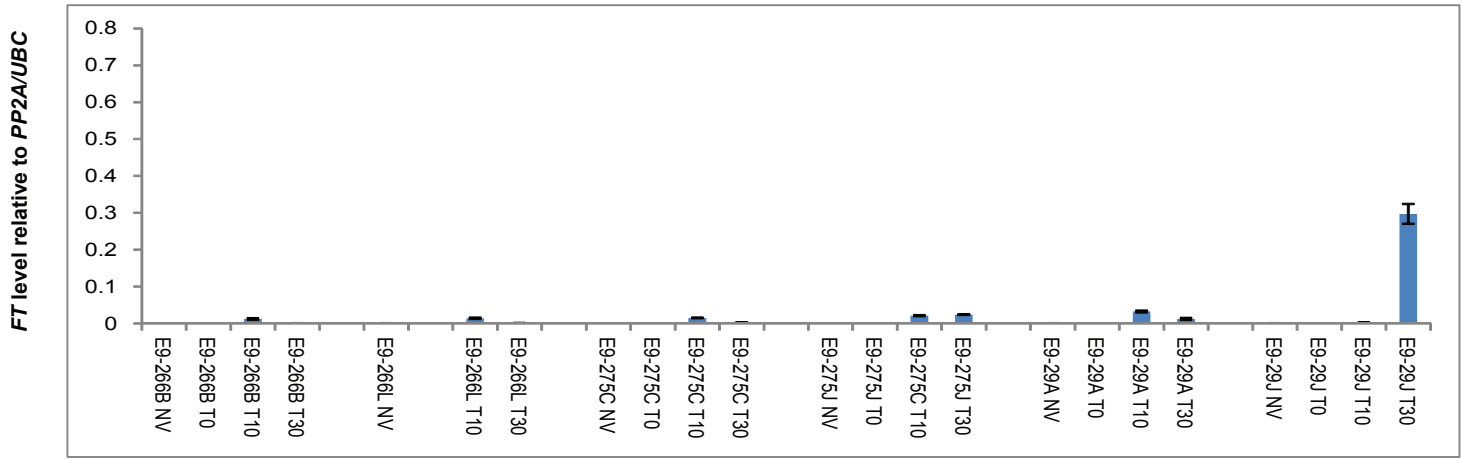

Supplement: Supplementary file 6 — Figure S6. Expression of AtFT.C2 in transgenic Arabidopsis. [file TPJ-87-597-s006.pdf]
