# Supplementary material for: Nucleotide polymorphism affecting FLC expression underpins heading date variation in horticultural brassicas
Source: Plant J. 2016 Jul 19;87(6):597–605. doi: 10.1111/tpj.13221 (PMC5053238; doi:10.1111/tpj.13221)

Supplementary figure 2.

(a)

Exon 2 spans positions 1321–1356.

Accession	Type	233	248	261	272	281	303	340	421	471	497	500	508	512	583	607	642	733-947	955	966	1050	1057	1058	1059	1072	1170	1183	1197	1219	1281	1286	1321	1324	1339	1354	1355	1356	Exon 4 SNP	
	E5 *BoFLC.C2*	C	G	G	T	G		T	G	*	T	C	A	A	C	T	G		A	A	A	A	*	*	A	A	T	A	A	G	G	G	A	G	G	A	T	N	*BoFLC.C2*$^{1-1}$
HRIGRU005293	Cauliflower	C	G	G	T	G		T	G	*	T	C	A	A	C	T	G		A	A	A	A	*	*	A	A	T	A	A	G	G	G	A	G	G	A	T	N/A	*BoFLC.C2*$^{1-1}$
HRIGRU006797	Autumn cauliflower	C	G	G	T	G		T	G	*	T	C	A	A	C	T	G		A	A	A	A	*	*	A	A	T	A	A	G	G	G	A	G	G	A	T	Y	*BoFLC.C2*$^{1-1}$
HRIGRU002398	Broccoli	C	G	G	T	G		T	G	*	T	C	A	A	C	T	G		A	A	A	A	*	*	A	A	T	A	A	G	G	G	A	G	G	A	T	Y	*BoFLC.C2*$^{1-1}$
HRIGRU006210	Fodder black kale	C	G	G	T	G		T	G	*	T	C	A	A	C	T	G		A	A	A	A	*	*	A	A	T	A	A	G	G	G	A	G	G	A	T	N	*BoFLC.C2*$^{1-1}$
HRIGRU002891	Winter cauliflower	C	G	G	T	G		T	G	*	T	C	A	A	C	T	G		A	A	A	A	*	*	A	A	T	A	A	G	G	G	A	G	G	A	T	N/A	*BoFLC.C2*$^{1-1}$
HRIGRU006254	Autumn cauliflower	C	G	G	T	G		T	G	*	T	C	A	A	C	T	G		A	A	A	A	*	*	A	A	T	A	A	G	G	G	A	G	G	A	T	Y	*BoFLC.C2*$^{1-1}$
HRIGRU008267	Kohl rabi	C	G	G	T	G		T	G	*	T	C	A	A	C	T	G		A	A	A	A	*	*	A	A	T	A	A	G	G	G	A	G	G	A	T	N/A	*BoFLC.C2*$^{1-1}$
HRIGRU004492	Winter cauliflower	C	G	G	T	G		T	G	*	T	C	A	A	C	T	G		A	A	A	A	*	*	A	A	T	A	A	G	G	G	G	G	G	A	T	N/A	*BoFLC.C2*$^{1-2}$
HRIGRU005443	Purple kohl rabi	C	G	G	T	G		T	G	*	T	C	A	A	C	T	A		A	A	A	A	*	*	A	A	T	A	A	G	G	G	A	G	G	A	T	N/A	*BoFLC.C2*$^{1-3}$
HRIGRU007833	Cabbage	C	G	G	T	G		T	G	*	*	C	G	*	T	T	G	DEL	G	A	T	T	T	G	G	G	C	A	T	T	T	A	G	A	*	*	*	N/A	*BoFLC.C2*$^{2-1}$
HRIGRU009836	Cabbage	T	G	G	T	G		T	G	*	*	C	G	*	T	T	G	DEL	G	A	T	T	T	G	G	G	C	A	T	T	T	A	G	A	*	*	*	N/A	*BoFLC.C2*$^{2-2}$
	E9 *BoFLC.C2*	T	T	A	T	A		T	G	*	*	C	G	*	T	T	G	DEL	G	A	T	T	T	G	G	G	C	A	T	T	T	A	G	A	*	*	*	N	*BoFLC.C2*$^{3-1}$
HRIGRU000605	Brussels sprout	T	T	A	T	A		T	G	*	*	C	G	*	T	T	G	DEL	G	A	T	T	T	G	G	G	C	A	T	T	T	A	G	A	*	*	*	N	*BoFLC.C2*$^{3-1}$
ECD15/148B	Kale	T	T	A	T	A		T	G	*	*	C	G	*	T	T	G	DEL	G	A	T	T	T	G	G	G	C	A	T	T	T	A	G	A	*	*	*	N	*BoFLC.C2*$^{3-1}$
HRIGRU006226	Kale	T	T	A	T	A		T	G	*	*	C	G	*	T	T	G	DEL	G	A	T	T	T	G	G	G	C	A	T	T	T	A	G	A	*	*	*	N/A	*BoFLC.C2*$^{3-1}$
GB24	Broccoli	T	T	A	T	A		T	G	*	*	C	G	*	T	T	G	DEL	G	A	T	T	T	G	G	G	C	A	T	T	T	A	G	A	*	*	*	N	*BoFLC.C2*$^{3-1}$
WOM38A	Cauliflower	T	T	A	T	A		T	G	*	*	C	G	*	T	C	G	DEL	A	A	T	T	T	G	G	G	C	A	T	T	T	A	G	A	*	*	*	Y	*BoFLC.C2*$^{3-2}$
SUR186a	Cauliflower	T	T	A	T	A		T	G	*	*	C	G	*	T	C	G	DEL	A	A	T	T	T	G	G	G	C	A	T	T	T	A	G	A	*	*	*	Y	*BoFLC.C2*$^{3-2}$
SIR5a	Cauliflower	T	T	G	C	A	INS	A	A	CT	*	T	A	*	C	T	G	DEL	A	G	T	T	T	G	G	G	C	G	T	T	T	A	G	A	*	*	*	N/A	*BoFLC.C2*$^{3-3}$
Cor12b	Broccoli	T	T	G	C	A	INS	A	A	CT	*	T	A	*	C	T	G	DEL	A	G	T	T	T	G	G	G	C	G	T	T	T	A	G	A	*	*	*	N/A	*BoFLC.C2*$^{3-3}$
HRIGRU005458	Autumn cauliflower	T	T	G	C	A	INS	A	A	CT	*	T	A	*	C	T	G	DEL	A	G	T	T	T	G	G	G	C	G	T	T	G	A	G	A	*	*	*	Y	*BoFLC.C2*$^{3-4}$

(b)

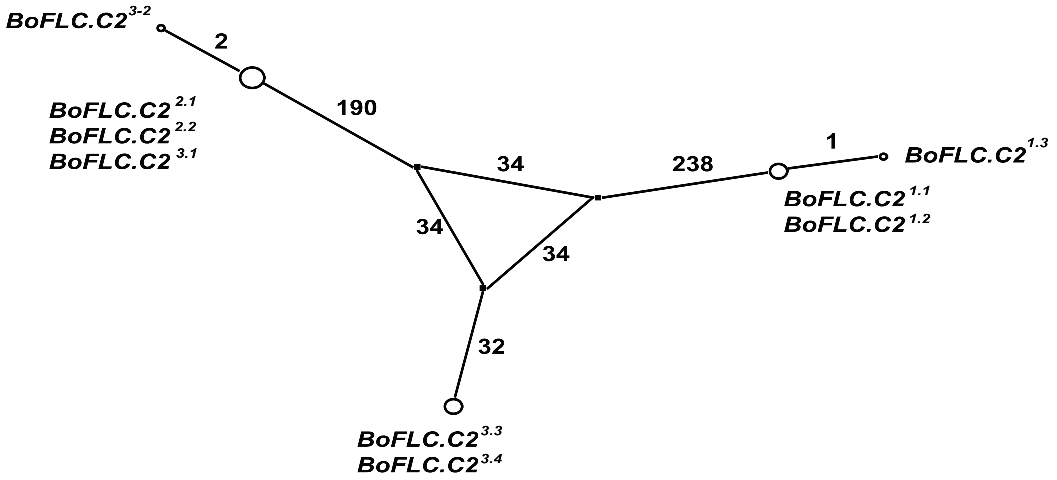

Supplement: Supplementary file 2 — Figure S2. Allelic variation at BoFLC.C2. [file TPJ-87-597-s002.pdf]
