# Supplementary material for: Nucleotide polymorphism affecting FLC expression underpins heading date variation in horticultural brassicas
Source: Plant J. 2016 Jul 19;87(6):597–605. doi: 10.1111/tpj.13221 (PMC5053238; doi:10.1111/tpj.13221)
Supplement: Supplementary file 8 [file TPJ-87-597-s008.docx]

**Legends for supporting Information**

**Supplementary Figure 1:** (a) Variation in heading date between four Broccoli genotypes grown at the Lincolnshire and Cornwall field sites in 2007/8 and 2011/12. Photographs have been edited to remove the large white labels.

(b) Histograms comparing heading dates of parental genotypes at the two field sites in Cornwall and Lincolnshire and showing consistent difference in heading date of the E5 and E9 parent plants under field conditions. Bars with the same letters are not significantly different when compared using a two-sided T-test (p<0.05) and multiple pairwise comparisons.

(c) Mean monthly temperatures at the field sites in 2007/8. Temperature data taken from the Met Office UK climate historical data (<http://www.metoffice.gov.uk/public/weather/climate-historic/#?tab=climateHistoric>)

(d) Heat map of QTL mapped to the nine *B. oleracea* linkage groups using single marker regression at two field independent field sites in Cornwall (CW) and Lincolnshire (LINC) in 2007/8 and 2011/12 and under glasshouse conditions following a 10 week vernalization under controlled conditions at 5^o^C and 10^o^C. CER= controlled environment room.

**Supplementary Figure 2:** Allelic variation at *BoFLC.C2*.

(a) Sample genotypes from each of the three major groups of alleles of which *BoFLC.C2^E5^* and *BoFLC.C2^E9^* represent the most common classes. Positions of polymorphisms are annotated with ATG as +1.

(b) Relationship between *FLC.C2* alleles analysed using SplitsTree4 software (Hudson and Bryant, 2006) using the median joining setting (Bandelt et al. 1999). The number along the branch shows the number of nucleotide differences.

**Supplementary Figure 3:** Expression analysis in E5 and E9 parent lines

(a) Left: *BoFLC.C2* expression in the E5 (dark grey) and E9 (light grey) parental genotypes following 10 weeks vernalization at 10^o^C. Error bars are standard errors of the mean. Right: *BoFLC.C2* expression expressed relative to the mean NV starting level for each allele. Relative expression levels are shown for the E5 (dark grey) and E9 (light grey) parental genotypes following 10 weeks vernalization at 10^o^C. Error bars are standard errors.

(b) Expression levels of the second *B.oleracea* orthologue of the floral promoter *FLOWERING LOCUS T* (*FT*) on chromosome C2. The graph shows *BoFT.C2* fold change relative to the starting level NV following 10 weeks at 5^o^C and 10^o^C. In both cases *BoFT.C2* is induced to a higher level at 14 days after return to warm conditions in E5 (dark grey) compared to E9 (light grey, p_5oC_ =0.007, p_10oC_=0.002 respectively). Error bars are standard errors.

**Supplementary Figure 4:** Flowering time of Arabidopsis BoFLC.C2 transgenics

(a) Histogram of flowering time of 62 *BoFLC.C2^E5^* and 39 *BoFLC.C2^E9^* independent Kanamycin resistant T1 lines without vernalization. Error bars are standard errors of the mean.

(b) Histogram of the mean flowering times of pools of five plants from two lines of each of the individual T_3_ families following 4, 6 and 8 weeks of vernalization at 5^o^C. Error bars are standard errors of the mean.

**Supplementary Figure 5:** Expression of *BoFLC.C2* alleles in transgenic Arabidopsis from pools of Arabidopsis seedlings of two individual lines for each of three homozygous T3 families expressing the *BoFLC.C2^E5^* and *BoFLC.C2^E9^* alleles following 4 weeks and 8 weeks vernalization at 5^o^C. Error bars are standard errors of the mean expression normalised to the geometric mean of *PP2A* and *UBC*.

**Supplementary Figure 6:** Expression of *AtFT* in transgenic Arabidopsis from pools of Arabidopsis seedlings of two individual lines for each of three homozygous T3 families expressing the *BoFLC.C2^E5^* and *BoFLC.C2^E9^* alleles following 4 weeks and 8 weeks vernalization at 5^o^C. Inset graph shows *AtFT* expression relative to *UBC* in *FRI flc2* non-transgenic controls following 4 weeks (4wV) and 8 weeks (8wV) vernalization. Error bars are standard errors of the mean, expression normalised to the geometric mean of *PP2A* and *UBC*.

**Supplementary Table 1:** Primers used in this study.
